# 

**Published:** 1887-02

**Authors:** 


### BOOK NOTICES.

Among the valuable monthlies which should have been included in our January issue are: The Peoples' Health Journal, of Chicago. Vick's Illustrated Monthly.

THE CENTURY for January, which came to hand after we had gone to press, is as full of good things as ever. The new chapter in the life of Abraham Lincoln, and the third day's battle of Gettysburg, are features which make it intensely interesting to every reader. We cannot say too much in commendation of this magazine, which well merits its enormous patronage.

VICK'S ILLUSTRATED MONTHLY MAGAZINE AND FLORAL GUIDE for January, 1887.—This is certainly all that could be desired in text and illustrations of a work devoted to flowers and their culture. We have seen nothing to surpass it.

